# Supplementary material for: Therapeutic plasma exchange for coronavirus disease-2019 triggered cytokine release syndrome; a retrospective propensity matched control study
Source: PLoS One. 2021 Jan 7;16(1):e0244853. doi: 10.1371/journal.pone.0244853 (PMC7790281; doi:10.1371/journal.pone.0244853)
Supplement: Attachment — Submitted filename: response to reviewers v 15 dec .2020.docx [file pone.0244853.s003.docx]

**Dated, 15^th^ December, 2020**

Dear
**Prof. Raffaele Serra, M.D., Ph.D
Academic Editor
PLOS ONE**

Manuscript Number: PONE-D-20-29085
Therapeutic plasma exchange for coronavirus disease-2019 triggered cytokine release syndrome; a retrospective propensity matched control study

Thank you for your email. We are pleased to provide a revised version of our manuscript. We want to thank the reviewers for their time and effort in reviewing our manuscript. We sincerely appreciate their comments and have endeavored to respond with an acceptable revision.

**Editor and Reviewer comments: (14/12/2020)**

**Additional Editor Comments:**

1. If the authors have adequately addressed your comments raised in a previous round of review and you feel that this manuscript is now acceptable for publication, you may indicate that here to bypass the “Comments to the Author” section, enter your conflict of interest statement in the “Confidential to Editor” section, and submit your "Accept" recommendation.

Reviewer #1: (No Response)

Answer: All comments raised in a previous round of review have been rectified please.

2. Is the manuscript technically sound, and do the data support the conclusions?

The manuscript must describe a technically sound piece of scientific research with data that supports the conclusions. Experiments must have been conducted rigorously, with appropriate controls, replication, and sample sizes. The conclusions must be drawn appropriately based on the data presented.

Reviewer #1: Partly

Answer: Thank you very much for giving us a chance to rectify this observation. The four females’ patients which were previously selected by PSM NCCS statistical software v20.0.2 have been replaced by four similar male patients having same age, comorbidities, duration of illness, symptoms, HRCT chest score, CRS markers, disease severity and other features. Three of them did not undergo TPE while TPE was performed in remaining one. Changes have been endorsed in conclusion section

3. Has the statistical analysis been performed appropriately and rigorously?

Reviewer #1: Yes

4. Have the authors made all data underlying the findings in their manuscript fully available?

The [PLOS Data policy](http://www.plosone.org/static/policies.action#sharing) requires authors to make all data underlying the findings described in their manuscript fully available without restriction, with rare exception (please refer to the Data Availability Statement in the manuscript PDF file). The data should be provided as part of the manuscript or its supporting information, or deposited to a public repository. For example, in addition to summary statistics, the data points behind means, medians and variance measures should be available. If there are restrictions on publicly sharing data—e.g. participant privacy or use of data from a third party—those must be specified.

Reviewer #1: Yes

5. Is the manuscript presented in an intelligible fashion and written in standard English?

PLOS ONE does not copyedit accepted manuscripts, so the language in submitted articles must be clear, correct, and unambiguous. Any typographical or grammatical errors should be corrected at revision, so please note any specific errors here.

Reviewer #1: Yes

6. Review Comments to the Author

Please use the space provided to explain your answers to the questions above. You may also include additional comments for the author, including concerns about dual publication, research ethics, or publication ethics. (Please upload your review as an attachment if it exceeds 20,000 characters)

Reviewer #1: Due to the single female patient in the TBE-treated group, it is unacceptable to state that "female gender was less represented in this study...therefore results of this trial cannot be generalized on females and gender based comparative analysis cannot be done."
Those 4 patient need to be removed from the entire analyses. Since this is a retrospective study, this can be easily done by removing the data points for those individuals.

*Answer: Thank you very much for giving us opportunity to revise our manuscript. As per your advice we have excluded four female patients from PSM cohort and replaced them with four similar male patients so that overall results not need to be recalculated from the beginning. We took extreme care to select very similar 4 male patients having same age, duration of symptoms, comorbidities, HRCT chest score and pattern, levels of CRS markers, oxygen support, disease severity and TPE procedure. If this is not acceptable to you then please let us know so that we can start recalculating and rewriting all results.*

7. PLOS authors have the option to publish the peer review history of their article ([what does this mean?](https://journals.plos.org/plosone/s/editorial-and-peer-review-process#loc-peer-review-history)). If published, this will include your full peer review and any attached files.


If you choose “no”, your identity will remain anonymous but your review may still be made public.


**Do you want your identity to be public for this peer review?** For information about this choice, including consent withdrawal, please see our [Privacy Policy](https://www.plos.org/privacy-policy).

Reviewer #1: No

Thank you so much for these very astute observations and your kind consideration. Your suggestions have clearly improved our manuscript. Please do not hesitate to contact us should you have any further concerns or questions, and we would be more than happy to address them. Please allow us to resubmit updated manuscript for review and consideration for publication.

Sincerely,

**Corresponding author for this manuscript is:**

Dr. Sultan Mehmood Kamran
